# MGF-DTA: A Multi-Granularity Fusion Model for Drug–Target Binding Affinity Prediction

**DOI:** 10.3390/ijms27020947

**Published:** 2026-01-18

**Authors:** Zheng Ni, Bo Wei, Yuni Zeng

**Affiliations:** School of Computer Science and Technology, Zhejiang Sci-Tech University, Hangzhou 310018, China; 2024220603061@mails.zstu.edu.cn (Z.N.); weibo@zstu.edu.cn (B.W.)

**Keywords:** drug–target affinity, multi-granularity fusion, hierarchical attention mechanism

## Abstract

Drug–target affinity (DTA) prediction is one of the core components of drug discovery. Despite considerable advances in previous research, DTA tasks still face several limitations with insufficient multi-modal information of drugs, the inherent sequence length limitation of protein language models, and single attention mechanisms that fail to capture critical multi-scale features. To alleviate the above limitations, we developed a multi-granularity fusion model for drug–target binding affinity prediction, termed MGF-DTA. This model is composed of three fusion modules, specifically as follows. First, the model extracts deep semantic features of SMILES strings through ChemBERTa-2 and integrates them with molecular fingerprints by using gated fusion to enhance the multi-modal information of drugs. In addition, it employs a residual fusion mechanism to integrate the global embeddings from ESM-2 with the local features obtained by the k-mer and principal component analysis (PCA) method. Finally, a hierarchical attention mechanism is employed to extract multi-granularity features from both drug SMILES strings and protein sequences. Comparative analysis with other mainstream methods on the Davis, KIBA, and BindingDB datasets reveals that the MGF-DTA model exhibits outstanding performance advantages. Further, ablation studies confirm the effectiveness of the model components and case study illustrates its robust generalization capability.

## 1. Introduction

Drug–target interaction (DTI) refers to the process where specific biological macromolecules within cells (the “targets”, primarily proteins such as enzymes and ion channels) bind to drug molecules with appropriate chemical properties and affinity [1]. In this process, the binding affinity can be used to characterize the interaction strength between drug–target pairs. The prediction of this affinity through experimental or computational methods is termed drug–target affinity (DTA) prediction. Determining the binding process between drug and target is crucial for understanding the mechanism of drug action. Therefore, DTA research has become one of the core components of drug discovery [2,3,4]. In addition, drug discovery is a time-consuming and costly process. A new medicine will take an average of 10–15 years and more than USD 2 billion before it can reach the pharmacy shelf [5]. Since the therapeutic effect of drugs often depends on their binding strength with the target protein, this also makes the study of DTA critically important. Early methods for DTA prediction were traditionally accomplished through wet-lab experiments, which are generally reliable and accurate but require strict conditions and have high costs [6]. Subsequently, classic machine learning approaches [7], such as random forest [8], support vector machines [9], and XGBoost [10,11], were introduced, incorporating shallow features of drugs and targets for prediction. In contrast, deep learning techniques not only demonstrate substantial advantages in prediction speed but also show remarkable potential in identifying complex bioinformatics patterns and extracting critical features [12].

In recent years, deep learning methods have been increasingly applied across various stages of drug development, with several efficient methods emerging. In this work, we focus on sequence-based DTA prediction without incorporating molecular graph representations, primarily because sequence models are computationally more scalable and efficient, thus enabling rapid experimentation and large-scale application. Initially, DeepDTA [13] utilized convolutional neural networks (CNNs) to learn features from drug SMILES strings and protein sequences. To further absorb more sequence information, WideDTA [14] proposed to integrate different pieces of text-based information to provide a better representation of the interaction. Subsequently, AttentionDTA [15] developed a novel two-side multi-head attention mechanism to explore the influence of attention calculation mode on model performance. In addition, TF-DTA [16] adopted transformer encoders and multi-layer CNNs to obtain better protein and drug representations. Furthermore, DCGAN-DTA [17] proposed a custom 1D deep convolutional generative adversarial network to extract features from drug SMILES and protein sequences, coupled with the introduction of evolutionary features via BLOSUM encoding and the use of an Add layer for feature fusion. More recently, PMMR [18] introduced an innovative approach that utilizes pre-trained models to extract drug and protein features separately, and employs a transformer for fine-tuning the extracted features.

Despite considerable advances in previous research, DTA tasks still face several limitations. First, the molecular feature representation remains incomplete. While existing methods like ChemBERTa can learn the deep semantic features of drug molecules, they fail to adequately capture the comprehensive chemical semantics underlying the structures. Second, the ESM model, constrained by its inherent transformer architecture, encounters severe computational bottlenecks when processing long sequences, with both memory and time costs growing quadratically with sequence length, making efficient processing difficult. Finally, most models rely on a single attention mechanism that tends to focus exclusively on the most prominent features in sequences, while overlooking other equally important features at different scales.

To alleviate the above limitations, we developed a multi-granularity fusion model for drug–target binding affinity prediction, termed MGF-DTA. This model is comprised of three fusion modules, specifically as follows. For drugs, we utilize ChemBERTa-2 to extract deep semantic features from SMILES strings and incorporate molecular fingerprint features, which are then combined via the gated fusion mechanism. This adaptive integration compensates for the lacking chemical information, thereby enhancing feature integrity. For proteins, we employ ESM-2 to extract global semantic features from sequences, while using a k-mer and principal component analysis (PCA) approach to obtain partial features. The residual fusion mechanism is then applied to integrate these representations, achieving effective complementarity between local and global information. To efficiently integrate drug and protein features, we introduce a hierarchical attention mechanism that progressively focuses on critical partial information at each layer and performs comprehensive judgment, thereby enabling precise predictions with interpretability.

The main contributions of our study are summarized as follows:

- We employ the ChemBERTa-2 model to extract deep semantic features from SMILES strings and perform gated fusion with traditional Morgan and Avalon fingerprints, thereby capturing overlooked chemical semantic information and constructing informative drug representations.
- To compensate for potential information loss caused by the sequence length limitations of the ESM-2 model, we employ a residual fusion mechanism to innovatively integrate features extracted through k-mer+PCA with ESM-2 features, thereby realizing complementarity between partial and global features.
- We design a hierarchical attention mechanism that computes independent attention distributions in parallel across multiple levels, achieving multi-granularity feature extraction from both drug SMILES strings and protein sequences.

## 2. Results and Discussion

In this section, we will discuss the performance of the MGF-DTA model in detail. The discussion can be divided into the following parts: (1) multi-modal feature enhancement for drug representation, (2) global–local feature complementarity for protein representation, (3) hierarchical attention fusion for DTA prediction, (4) performance comparison between MGF-DTA and other mainstream methods, (5) cross-domain generalization testing, (6) ablation experiment, (7) interpretability analysis, (8) case study, and (9) discussion. All results in the following table are marked with statistical significance.

### 2.1. Multi-Modal Feature Enhancement for Drug Representation

To construct a more comprehensive drug representation, we adopted a multi-feature fusion strategy that integrates the deep semantic features extracted by the ChemBERTa-2 model with two fingerprint features, Morgan and Avalon. Table 1 compares the performance of MGF-DTA with and without fingerprint features, demonstrating their impact on model effectiveness. Statistical analysis using Tukey HSD tests confirmed that the ChemBERTa-2+fingerprints method with gated fusion significantly outperformed the baseline ChemBERTa-2 method across most datasets and evaluation metrics (*p* < 0.05).

**Figure 1:**
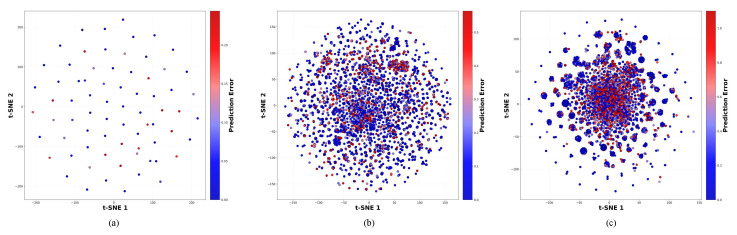
The t-SNE graphs show the clustering effect of the Morgan fingerprint (ECFP). (**a**) Davis dataset, (**b**) KIBA dataset, and (**c**) BindingDB dataset.

In Figure 1, we visualize the t-SNE graphs of the Morgan fingerprint (ECFP) for the Davis, KIBA, and BindingDB datasets. We can observe a certain clustering effect, which demonstrates the effectiveness of MGF-DTA.

Subsequently, we investigated various fusion methods for drug features to identify the most suitable approach for integrating ChemBERTa-2 features, Morgan fingerprints, and Avalon fingerprints. The evaluated methods include Concat, Weighted Fusion, Cross-Attention, and gated fusion. The results demonstrate that gated fusion outperforms all other methods across all evaluation metrics on the three benchmark datasets. This finding indicates that gated fusion can more effectively capture and leverage the dynamic complementary relationships among multi-modal information. The detailed results are presented in Table 2.

We employed one-way ANOVA followed by Tukey HSD tests for pairwise comparisons. Statistical significance is indicated using compact letter display (CLD).

### 2.2. Global–Local Feature Complementarity for Protein Representation

To overcome the length constraint of the ESM-2 model, we propose a feature extraction strategy based on k-mer frequency analysis combined with Principal Component Analysis (PCA). Table 3 containing significant markers compares the performance of MGF-DTA with and without k-mer features, unveiling their impact on model effectiveness.

**Figure 2:**
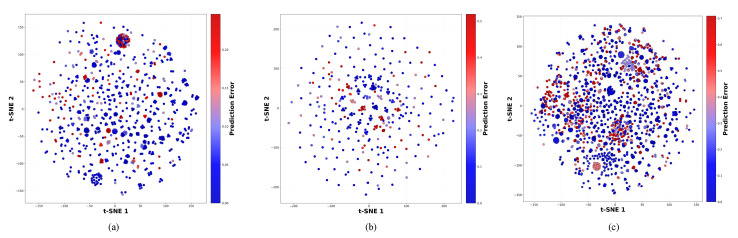
The t-SNE graphs show the clustering effect of the ESM-2 embeddings. (**a**) Davis dataset, (**b**) KIBA dataset, and (**c**) BindingDB dataset.

In Figure 2, we visualize the t-SNE graphs of the ESM-2 embeddings for the Davis, KIBA, and BindingDB datasets. We can observe a certain clustering effect, which proves the effectiveness of MGF-DTA.

### 2.3. Hierarchical Attention Fusion for DTA Prediction

We investigated various fusion methods for drug–target features to identify the most suitable approach for integrating drug and protein representations. The evaluated methods include Concat, Bilinear Fusion, Weighted Fusion, Cross-Attention Fusion, Linear Attention Fusion, and Hierarchical Attention Fusion. The results show that Hierarchical Attention Fusion outperforms all other methods across every evaluation metric on the three benchmark datasets. This finding indicates that Hierarchical Attention Fusion can more effectively integrate multi-scale information from both drugs and proteins, thereby enabling more accurate prediction of their binding affinity and bioactivity. The detailed results using compact letter display (CLD) are presented in Table 4.

### 2.4. Performance Comparison Between MGF-DTA and Other Mainstream Methods

We conducted a comprehensive performance comparison between MGF-DTA and other mainstream methods, including DeepDTA [13], AttentionDTA [15], TF-DTA [16], PMMR [18], GraphDTA [19], MGraphDTA [20], LLMDTA [21], and SMFF-DTA [22], under consistent experimental settings across two benchmark datasets. The detailed results are shown in Table 5.

In the evaluation on the Davis dataset, our model demonstrates superior performance in the MSE, CI, and $R_{m}^{2}$ metrics. Notably, compared to the next best performing PMMR method, MGF-DTA reduces MSE by 5.67%, increases CI by 0.329%, and improves $R_{m}^{2}$ by 3.73%. Furthermore, MGF-DTA shows significant differences across all evaluation metrics when compared to the aforementioned mainstream methods (*p* < 0.05).

In the KIBA dataset, MGF-DTA outperforms all mainstream methods across all metrics. Notably, compared to the next best performing MGraphDTA method, MGF-DTA achieves a 10.81% reduction in MSE and improvements of 0.335% in CI and 1.16% in $R_{m}^{2}$. Moreover, MGF-DTA is statistically significantly superior (*p* < 0.05) to the mainstream methods in terms of MSE, CI, and $R_{m}^{2}$.

Since the BindingDB dataset was preprocessed from the 2020 version, we conducted a comparative analysis solely against the next best performing PMMR method. The detailed results are presented in Table 6.

In the BindingDB dataset, compared to the PMMR method, MGF-DTA reduces MSE by 2.703% and increases CI by 0.459% and $R_{m}^{2}$ by 0.658%. Furthermore, MGF-DTA significantly outperforms PMMR across all metrics (*p* < 0.05).

**Figure 3:**
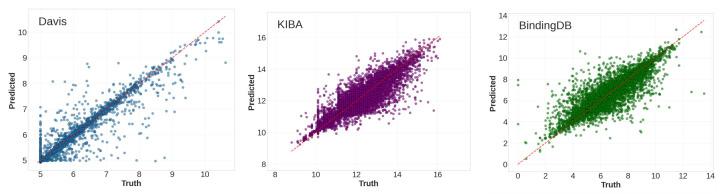
Ground truth affinities (*x*-axis) vs. predicted affinities (*y*-axis) for drug–target pairs in Davis, KIBA, and BindingDB datasets.

To further analyze the experimental results, we plotted the predicted binding affinity against the ground truth for the Davis, KIBA, and BindingDB datasets. Figure 3 shows the corresponding scatter plots. With the ground truth values on the *x*-axis and the predicted values on the *y*-axis, an ideal model would produce points lying directly on the line y = x. As shown in Figure 3, the data points are situated on or near this line and are distributed symmetrically around it. Furthermore, the results indicate that our model achieved superior performance on the KIBA dataset, as the points were more densely distributed around the ideal line y = x.

### 2.5. Cross-Domain Generalization Testing

In this section, the performance of MGF-DTA was evaluated on the Therapeutics Data Commons Domain Generalization (TDC-DG) dataset. This dataset divides the collected affinity data into training and test sets based on year, making it less likely for data from the training set to leak into the test set and thereby placing higher demands on the model’s generalization capability. As shown in Table 7, compared with other methods, our proposed approach MGF-DTA achieves the most significant results on the Pearson index.

### 2.6. Ablation Experiment

To demonstrate the necessity of the fusion module, we tested the impact of different fusion methods on model performance. Specifically, we selected Model-1 as the baseline method, whose primary inputs were ChemBERTa-2 features and ESM-2 features. We then enhanced the baseline by gradually adding the gated fusion of drugs, residual fusion of proteins, and the hierarchical attention mechanism, corresponding to Model-2, Model-3, and Model-4, respectively. The results of the baseline and the newly configured models are presented in Table 8. Model-2 employs the gated fusion mechanism for integrating the drug’s fingerprint features with ChemBERTa-2 features. This approach allows the model to dynamically weight each drug feature representation based on their relevance to the specific protein target, resulting in adaptive and context-aware drug embeddings. Based on the results from Model-3, the residual fusion effectively preserves the original protein sequence information while incorporating complementary information from the k-mer features. The performance of Model-4 is further enhanced by integrating the above-mentioned drug and protein features through the hierarchical attention mechanism. Specifically, on the Davis dataset, Model-4 achieves an MSE of 0.183 and a CI of 0.913, substantially outperforming the baseline method. This improvement can be attributed to its ability to compute multiple attention distributions in parallel, enabling the model to capture diverse information across different hierarchical levels of the sequences. In summary, the combined effect of all proposed innovative components surpasses the baseline method, thereby fully validating the superiority of the individual design elements.

### 2.7. Interpretability Analysis

Traditional deep learning methods for drug–target affinity prediction are often “black box” models, making their internal decision-making processes difficult to understand. With the development of the attention mechanism, it becomes possible to convert from black box mode to white box mode. In this study, the hierarchical attention mechanism incorporated in MGF-DTA allows us to investigate drug–target interaction mechanisms by analyzing attention weights.

**Figure 4:**
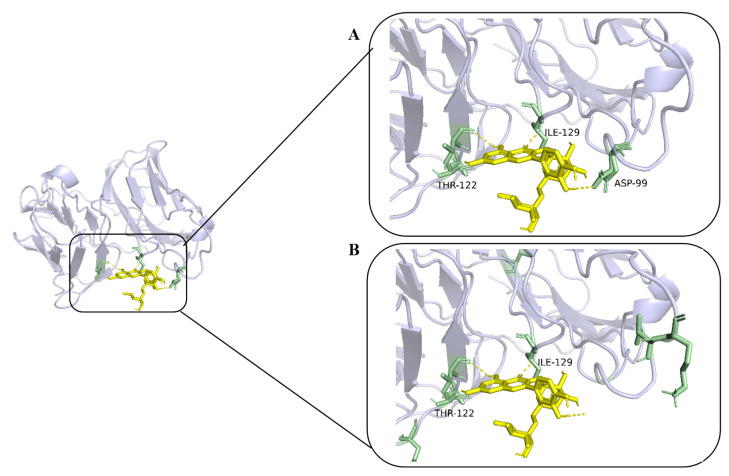
The visualization of interaction sites in 2Q8O. (**A**) Potential interaction sites marked in green. (**B**) Predicted interaction sites marked in green.

We chose 2Q8O in the Protein Data Bank (PDB) database as a case for weight visualization. We selected amino acid residues with higher attention weight values as interaction sites predicted by MGF-DTA, and the number of selected amino acid residues is equal to the number of potential interaction sites. As illustrated in Figure 4, both the potential and predicted interaction sites for 2Q8O are highlighted in green. The model accurately identified potential binding sites within the binding pockets THR-122 and ILE-129. However, it also ignored the ASP-99 binding site. Despite these occasional inaccuracies in designating binding regions, MGF-DTA demonstrates an ability to prioritize residues with binding potential, suggesting that it has some interpretability for exploring drug–target pairs.

### 2.8. Case Study

During the model evaluation phase, to examine its cross-dataset generalization capability, we conducted an external validation using 15 compound samples specifically selected from the Metz dataset. The Metz dataset is derived from biochemical assay-based interaction information and exhibits notable differences in data distribution and feature dimensions compared to commonly used training sets such as Davis, KIBA, and BindingDB. Therefore, it serves as a suitable independent test set for assessing the model’s adaptability to novel structural data. To ensure the rigor of the test, we deliberately excluded any protein–ligand pairs that appeared in the Davis, KIBA, or BindingDB datasets during the sample selection process. This step completely avoids evaluation bias caused by training data leakage and guarantees that the selected samples are entirely unseen by the model, thereby reflecting the model’s true predictive performance when encountering novel complexes in real-world scenarios. Furthermore, since the KIBA dataset not only integrates multi-source bioactivity data but also offers richer interaction information and larger data scale, we chose to use the model trained on KIBA for prediction, with the expectation of leveraging the broader binding patterns it has learned. The detailed results are presented in Table 9.

From the table, we observed that the predicted rankings for 13 out of the 15 samples aligned with the true values from the Metz dataset. To some extent, this indicates that our proposed model possesses good generalization capability. Even when confronted with entirely new protein–ligand pairs not encountered during training, the model maintained predictive consistency in the majority of cases.

Regarding the erroneous samples, we believe that the KIBA-trained model has its own inherent limitations, possibly arising from differences in data distributions. Nevertheless, this observation points toward a potential direction for future improvements, such as investigating how to better process sequences to enhance the universality of the model.

### 2.9. Discussion

From the perspective of drug discovery, the core value of the MGF-DTA model lies in its multi-level intelligent information fusion, which provides effective solutions for predicting drug–target interactions of varying difficulty. Specifically, targets such as kinases—particularly their Type I inhibitors—are generally considered relatively easier to predict, as their binding pockets (e.g., ATP-binding sites) are evolutionarily conserved, and the chemical patterns of their ligands are relatively regular. The model’s adaptability to such scenarios is reflected in several aspects: it leverages ESM-2 to extract deep information about conserved domains and functional sites from protein sequences, while reinforcing the capture of key local motifs (e.g., the DFG motif) through 3-mer features. At the same time, drug molecules are represented through contextualized features learned by ChemBERTa-2 and explicit substructure information encoded by Morgan fingerprints, which are integrated via a gated fusion mechanism to form robust representations. However, for targets with more complex binding mechanisms, such as Type II inhibitors that bind to the inactive conformation (DFG-out) of kinases, the predictive challenge lies in indirectly inferring the conformational plasticity of the protein. The model attempts to capture sequence signals related to conformational changes through the residual fusion of global ESM-2 features and local 3-mer features, though this remains an inherent challenge when predicting from one-dimensional sequences alone.

A more representative challenge is posed by G protein-coupled receptors (GPCRs), where the difficulty fundamentally stems from the ability of a single receptor sequence to adopt multiple conformational states (active/inactive), and ligands with different functions (e.g., agonists and antagonists) can stabilize specific states. The model’s potential to address this complexity lies in the following process: ESM-2 may implicitly encode topological constraints and conserved residue information of GPCR transmembrane helices, providing a basis for inferring conformational preferences. The hierarchical attention mechanism may learn interaction patterns between specific drug substructures and specific protein residues, which could correlate with conformational states. Nevertheless, the model still cannot provide explicit three-dimensional conformational information, representing a fundamental limitation. Therefore, for experimentalists, the model offers high-accuracy predictions for targets with well-defined binding modes, while for flexible targets like GPCRs, it maximizes the extraction of implicit correlations from sequences and chemical structures.

## 3. Materials and Methods

### 3.1. Model Architecture

**Figure 5:**
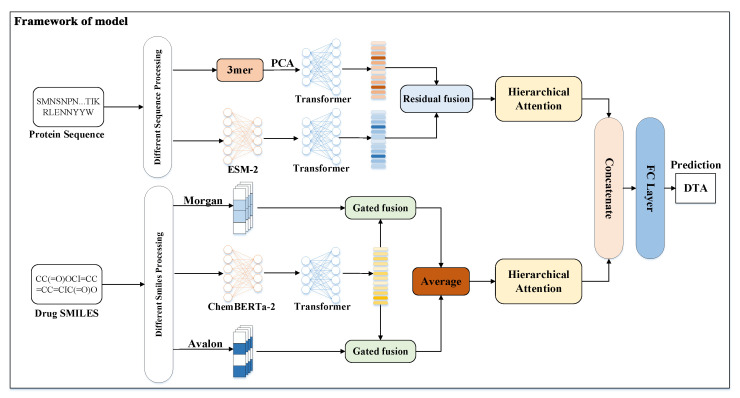
The overall architecture of MGF-DTA.

The overall architecture diagram of MGF-DTA is shown in Figure 5. For drug molecules, we first utilize the ChemBERTa-2 pre-trained model to extract deep contextual features from the SMILES strings. Additionally, the Rdkit tool is employed to generate Morgan and Avalon fingerprints as additional information. The ChemBERTa-2 features are separately fused with two types of fingerprints through the gated fusion mechanism, followed by averaging of the two fused representations to form the comprehensive drug feature representation. For proteins, we employ the ESM-2 pre-trained model to extract high-dimensional embeddings from the protein sequences. To mitigate information loss caused by sequence length limitations of ESM-2, we also generate embeddings for the full protein sequence by using the 3-mer+PCA method, which are then processed by a transformer encoder to capture features. Subsequently, the partial 3-mer features and the global ESM-2 features undergo residual fusion to produce the overall protein feature representation. Next, drug and protein features are separately processed through a hierarchical attention mechanism to extract effective features independently. Finally, the extracted features are concatenated and fed into fully connected layers for affinity prediction.

#### 3.1.1. Drug Encoding

In the drug encoding section, we employ the chemical language model ChemBERTa-2 (MLM) [23] to obtain pre-trained features of drug SMILES strings. ChemBERTa-2 is a BERT-like transformer model that learns molecular fingerprints through semi-supervised pre-training of the language model. ChemBERTa-2 employs masked-language modeling (MLM) and multi-task regression (MTR) over a large corpus of 77 million SMILES strings, a well-known text representation of molecules. The generated feature representation is as follows:

(1) $$c_{smi}=\mathrm{ChemBERTa}\left(X_{smi}\right)W_{d}\in R^{n\times d_{c}}$$

where n is the length of SMILES strings, $d_{c}$ is the dimension of the hidden layer, and $W_{d}$ is the trainable weight matrix.

To ensure consistent SMILES lengths across each batch, we use the maximum SMILES length as a uniform length. Subsequently, a transformer is employed to fine-tune the pre-trained SMILES features.

(2) $$f_{s}=\mathrm{transformer}\left(c_{smi}\right)$$

Furthermore, drug SMILES strings can also be expressed through Morgan fingerprints and Avalon fingerprints. The Morgan fingerprint, also known as the Extended Connectivity Fingerprint (ECFP), is a topology-based “circular fingerprint” that effectively captures the local chemical environment around atoms and is highly sensitive to identifying key pharmacophores. Morgan fingerprint features are represented as $f_{mor}$.

The Avalon fingerprint is generated using its dedicated toolkit and does not rely on iterative circular expansions. Each bit directly corresponds to a specific chemical substructure, enabling it to not only effectively capture molecular geometric and orientation features but also exhibit excellent interpretability. Avalon fingerprint features are represented as $f_{ava}$.

#### 3.1.2. Drug Feature Fusion

**Figure 6:**
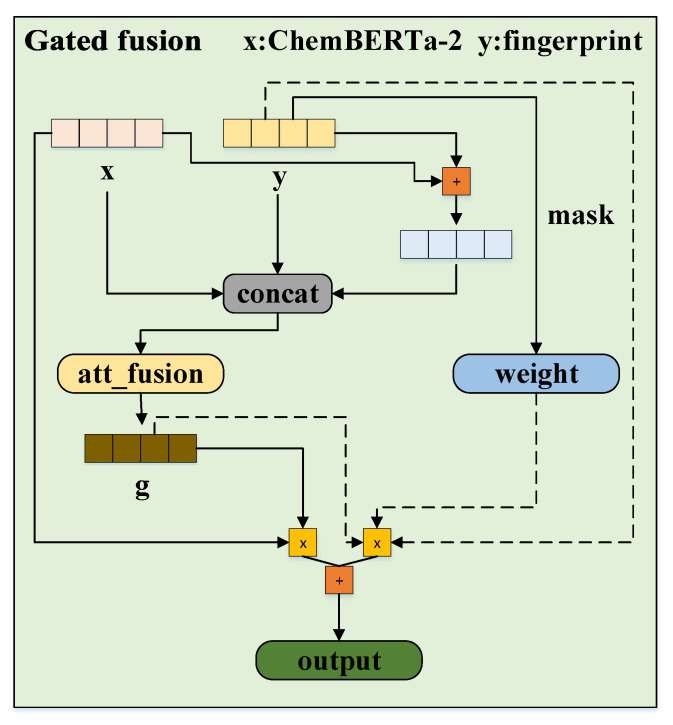
The workflow of gated fusion.

As shown in Figure 6, we first normalize the ChemBERTa-2 features and apply dimensionality reduction to the molecular fingerprints to unify their feature dimensions. Since Morgan and Avalon fingerprints are fused with drugs through gating, we will use the Morgan fingerprint as an example to illustrate the operational procedure in the following section.

First, the drug SMILES features, the Morgan fingerprint features, and their summation result are concatenated:

(3) $$Z=\mathrm{Concat}[f_{s},f_{mor},f_{s}+f_{mor}]$$

Fusion weights with position awareness are learned through the gated network:

(4) $$G=\sigma (W_{g}\cdot Z+b)$$

where $W_{g}$ is the learnable parameters and σ is the sigmoid activation function. Additionally, we introduce a feature-level attention mechanism to dynamically evaluate the importance of features at each position. The initial weight formula is defined as follows:

(5) $$E=\frac{f_{s}⊙f_{mor}}{\sqrt{d}}$$

where ⊙ denotes element-wise dot product, and d is the scaling factor. A sequence mask is then applied to handle padded positions, followed by Softmax normalization to obtain the attention weights, which are subsequently averaged:

(6) $$\left\{\begin{array}{c} A=\mathrm{Softmax}(\mathrm{Mask}(E,M))\\ W_{f}=\mathrm{mean}\left(A\right) \end{array}\right.$$

Finally, the fused feature representation is obtained as follows:

(7) $$f_{d}^{\prime }=G\cdot f_{s}+\left(1-G\right)\cdot f_{mor}\cdot W_{f}$$

The ChemBERTa-2 features are then fused with the Avalon fingerprint following a similar gated fusion process as described above. The average of the two fused features is taken to obtain the final feature representation $f_{d}$.

#### 3.1.3. Protein Encoding

For the processing of protein sequences, we utilize the protein language model ESM2-35 [24] to generate the initial feature representation. ESM-2 is a protein language model based on the transformer architecture. Pre-trained on large-scale protein sequence databases, it is capable of extracting deep representations directly from individual amino acid sequences and can be used to predict structural, functional, and evolutionary information of proteins. The initial feature representation extracted by ESM-2 is as follows:

(8) $$e_{p}=\mathrm{ESM}\left(P_{s}\right)W_{p}\in R^{n\times d_{t}}$$

where n is the sequence length, $d_{t}$ is the hidden layer dimension, and $W_{p}$ is a trainable weight matrix.

To adapt the initial features for downstream task requirements, we fine-tune the pre-trained features by using a transformer. Additionally, since protein sequences vary in length, we standardize them by taking the maximum length within each batch as the uniform length. The generated feature representation is as follows:

(9) $$f_{p}=\mathrm{transformer}\left(e_{p}\right)$$

**Figure 7:**
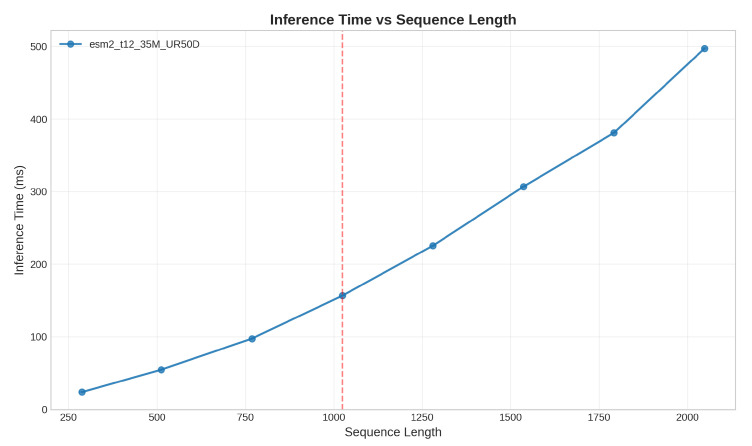
Inference time scaling with sequence length in ESM-2. The red line represents the maximum sequence length that ESM-2 can handle.

**Figure 8:**
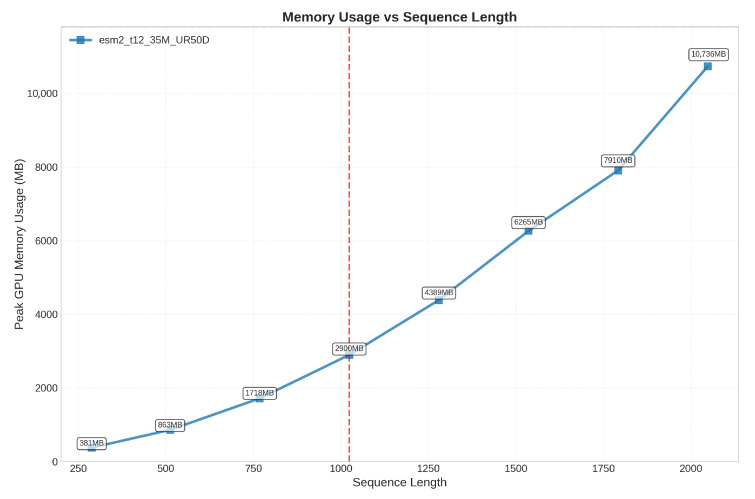
Peak memory usage scaling with sequence length in ESM-2. The red line represents the maximum sequence length that ESM-2 can handle.

Additionally, the transformer architecture adopted by ESM-2 has an inherent sequence length limitation, typically handling a maximum of 1024 residues. This constraint mainly arises from the O(n^2^) computational complexity required by the self-attention mechanism, making it difficult to efficiently process longer protein sequences. Based on this limitation of ESM-2, we further investigate the actual performance of GPU inference time and memory usage across different sequence lengths. Through systematic testing of the ESM-2 model with a batch size of 16, processing protein sequences ranging from short ones to those approaching the maximum length (2048 residues), we observe that GPU inference time and peak memory usage exhibit an approximately quadratic growth trend as sequence length increases. This directly confirms the memory bottleneck caused by the O(n^2^) computational complexity of the self-attention mechanism. The detailed results are presented in Figure 7 and Figure 8.

To overcome the length constraint of ESM-2 and capture the features of long sequences, we propose a feature extraction strategy based on k-mer frequency analysis combined with Principal Component Analysis (PCA). In this work, the k-value is set to 3, a choice that sufficiently captures protein sequence information while avoiding dimensionality explosion.

First, protein sequences are partitioned into contiguous subsequences of length 3 by using a sliding window approach [25]. For a protein sequence S = $a_{1}a_{2}\ldots a_{L}$ of length L, a sliding window of size 3 and stride 1 is applied to traverse the entire sequence, generating all possible 3-mer fragment sets K={S[i:i+3]∣i=1,2,…,L−2}. These fragments capture local amino acid compositional patterns and short-range correlation features within the protein sequence.

Subsequently, the frequency of each unique 3-mer fragment across the entire sequence is counted to construct a high-dimensional frequency feature vector. During processing, due to the vast amount of protein sequence data, the number of unique k-mers generated can become extremely large, theoretically up to $20^{k}$ possibilities. This leads to a rapid increase in feature dimensionality, imposing enormous computational and storage burdens. To address this, we construct a limited vocabulary of 3-mer features, controlling the feature dimension by setting a maximum vocabulary size. The frequency vectors are normalized by converting absolute counts into relative frequencies, thereby eliminating bias brought by sequence length variation. Assume there are three protein sequences S1: MATSK; S2: MATTE; S3: CATSE. Performing 3-mer extraction on the above sequence, we can obtain S1: [MAT, ATS, TSK]; S2: [MAT, ATT, TTE]; and S3: [CAT, ATS, TSE]. Based on the above sequences, retaining N = 7 distinct 3-mers as the feature vocabulary results in [MAT, ATS, TSK, ATT, TTE, CAT, TSE]. Next, the count of each k-mer is divided by the total number of k-mers in the corresponding sequence to obtain relative frequencies. The resulting vectors are S1 vector: [0.333, 0.333, 0.333, 0, 0, 0, 0]; S2 vector: [0.333, 0, 0, 0.333, 0.333, 0, 0]; S3 vector: [0, 0.333, 0, 0, 0, 0.333, 0.333]. Finally, the vectors of all sequences are stacked to form a matrix A.

To extract the most discriminative information from the high-dimensional k-mer frequency features, Principal Component Analysis (PCA) [26] is employed for dimensionality reduction. Before dimensionality reduction, Z-score standardization [27] is applied to ensure all feature dimensions are on the same scale. The Z-score standardization formula is shown below:

(10) $$Z_{ij}=\frac{A_{ij}-\mu_{j}}{\sigma_{j}}$$

where $A_{ij}$ represents the relative frequency of the j-th 3-mer in the i-th sample, $\mu_{j}$ is the mean of feature j, and $\sigma_{j}$ is the standard deviation of feature j. PCA dimensionality reduction primarily projects high-dimensional data into a low-dimensional space through linear transformation. First, the covariance matrix is computed and eigenvalue decomposition is performed:

(11) $$\left\{\begin{array}{c} C=\frac{1}{n-1}Z^{T}Z\\ Cv=\lambda v \end{array}\right.$$

where v is the eigenvector (principal component direction) and λ is the eigenvalue (variance size). Next, the eigenvalues are sorted in descending order, and the top k eigenvectors are selected. Suppose the eigenvalues are [3.2, 2.1, 0.8, 0.5, 0.3, 0.1, 0] and the corresponding eigenvectors are [*v*_1_, *v*_2_, *v*_3_, *v*_4_, *v*_5_, *v*_6_, *v*_7_]. Finally, the data is projected into the low-dimensional space:

(12) $$e_{mer}=Z\cdot V_{k}$$

where $V_{k}$ is the matrix composed of the top k eigenvectors.

In practical applications, we adopt a PCA dimensionality reduction method based on a preset dimension, uniformly reducing the k-mer frequency features to 1024 dimensions. This approach retains the top 1024 principal components that contribute the most variance, prioritizing the preservation of the primary variation patterns in the sequences during the reduction process. Subsequently, the dimensionality-reduced 3-mer features are fed into a transformer module for feature extraction. Finally, an attention pooling layer outputs the final 3-mer features, as shown below.

(13) $$f_{mer}=\mathrm{transformer}\left(e_{mer}\right)$$

#### 3.1.4. Protein Feature Fusion

**Figure 9:**
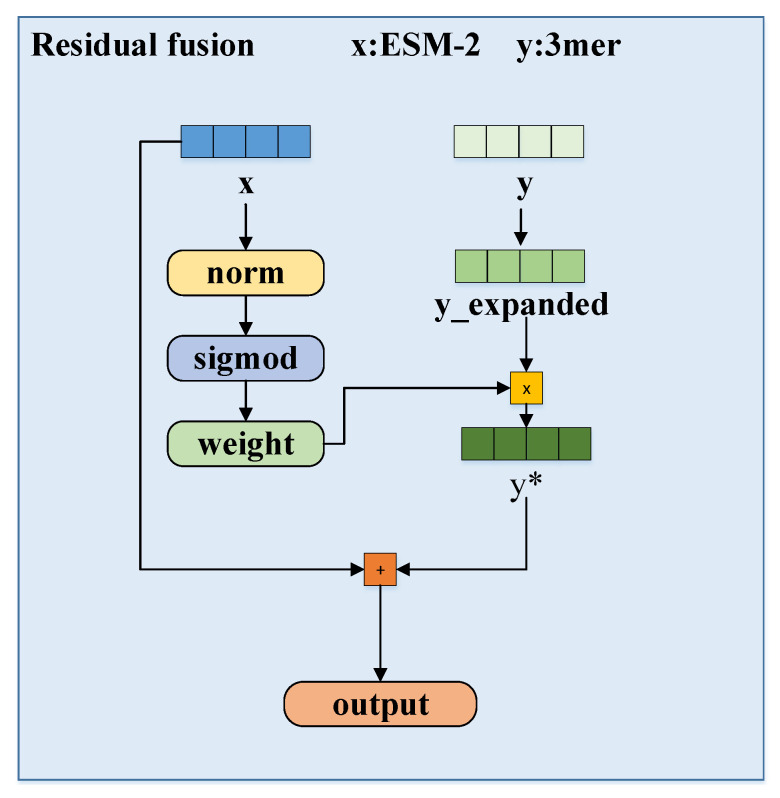
The workflow of residual fusion.

As shown in Figure 9, to prevent feature distribution shift, the ESM-2 features and 3-mer features of proteins are normalized separately.

Subsequently, the contribution of the 3-mer features is dynamically adjusted based on the confidence of the ESM-2 features: when the norm of the ESM-2 features is small, the weight of the 3-mer features increases, and vice versa. The initial confidence formula is given below.

(14) $$W_{esm}^{\prime }=\sqrt{\sum_{d=1}^{D}x_{i,j,d}^{2}}$$

where i is the batch index, j is the sequence position index, d is the feature dimension index, and D is the total feature dimension. The confidence is normalized to the interval (0,1) via a sigmoid function to obtain the final confidence score $W_{esm}$. Finally, the original ESM-2 features are combined with the adaptively weighted 3-mer features through a residual connection, obtaining the final protein feature representation:

(15) $$f_{t}=f_{p}+\left(1-W_{esm}\right)\cdot f_{mer}$$

#### 3.1.5. Hierarchical Attention Fusion and DTA Prediction

**Figure 10:**
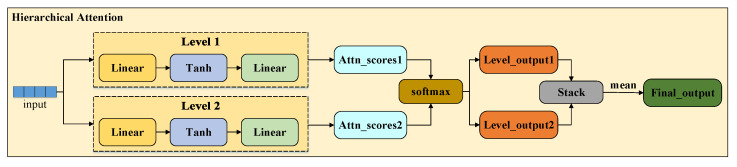
The workflow of the hierarchical attention mechanism.

To obtain effective feature representations and explore the interaction mechanisms between drugs and proteins, the features of drugs and proteins are passed into a hierarchical attention mechanism, as shown in Figure 10.

The computation of single-layer attention is performed as follows:

(16) $$S_{i}=W_{i,2}\tanh\left(W_{i,1}X\right)$$

To ensure that the model focuses only on valid sequence positions, a masked mechanism is applied to handle padded positions, and the attention scores are normalized via the Softmax function:

(17) $$A_{i}=\mathrm{Softmax}\left(S_{i}^{masked}\right)$$

Subsequently, the outputs from each level are computed and aggregated through average pooling:

(18) $$\left\{\begin{array}{c} C_{i}=A_{i}*X\\ C_{fin}=\frac{1}{N}\sum_{i=1}^{N}C_{i} \end{array}\right.$$

For both drugs and proteins, hierarchical attention is employed to extract effective features:

(19) $$\left\{\begin{array}{c} F_{d}=\mathrm{HierarchicalAttention}\left(f_{d}\right)\\ F_{t}=\mathrm{HierarchicalAttention}\left(f_{t}\right) \end{array}\right.$$

Finally, the obtained features are fed into a fully connected layer for DTA prediction, as shown below.

(20) $$y_{pre}=\mathrm{FC}\left(\mathrm{Concat}\left(F_{d},F_{t}\right)\right)$$

### 3.2. Experiment Setting

In this study, three publicly available benchmark datasets—Davis [28], KIBA [29], and BindingDB [30]—were used to evaluate the performance of MGF-DTA. The Davis dataset comprises 68 drugs and 442 kinase proteins, forming a total of 30,056 drug–target pairs. Their affinity values were assessed by using experimentally determined $K_{d}$ values. The KIBA dataset is larger in scale, containing approximately 246,000 interactions between 2111 drugs and 229 targets, with KIBA scores employed as the affinity metric. For the BindingDB dataset, we utilized the training and test sets from the 2020 release and performed the following preprocessing steps on the raw data: excluding multi-chained protein, excluding the items that do not have a UniprotID, excluding the items that do not have affinity label, choosing the higher affinity label when there are two pairs of the same drug–target, transforming the affinity label to float, and performing this transformation 9-lg(affinity). In addition, the BindingDB dataset adopts $K_{d}$ values as the affinity indicator.

Compared to existing fusion methods, our MGF-DTA model introduces three key distinctions. First, for drugs, we integrate interpretable molecular fingerprints with deep semantic features from SMILES strings through the gated fusion mechanism, exceeding the graph features used in PMMR to incorporate explicit chemical prior knowledge. Second, for proteins, we augment the global semantic features from ESM-2 with efficiently captured local features via the k-mer+PCA method, unlike PMMR which relies solely on pre-trained embeddings, thereby achieving a more comprehensive representation. Finally, for interaction modeling, we employ a hierarchical attention mechanism that progressively focuses on key substructures across multiple layers, providing finer-grained integration compared to the single-stage attention in AttentionDTA.

In previous DTA studies, benchmark datasets were typically divided into training and test sets. To more accurately reflect the practical performance of models and enhance experimental stability, this study divides the benchmark datasets into training, validation, and test sets at an 8:1:1 ratio. For the Davis, KIBA, and BindingDB datasets, we use 5 × 5 repeated cross-validation to generate 25 performance metric samples for our model. The entire training process is set to use the Adam optimizer to optimize parameters, and use the ReduceLROnPlateau function to dynamically adjust the learning rate.

We try to design different learning rates (lr), batch sizes, and epochs for each dataset and use grid search to determine the best parameters. We tried 1 × 10^−1^, 1 × 10^−2^, 1 × 10^−3^, 1 × 10^−4^, 5 × 10^−2^, 5 × 10^−3^, 5 × 10^−4^ as learning rates, and finally selected 1 × 10^−3^, 1 × 10^−3^, 1 × 10^−3^ for training and testing on the Davis, KIBA, and BindingDB datasets, respectively, as this learning rate demonstrated stable convergence during training and achieved better performance on the test set compared to other candidate values. For the epochs of Davis, KIBA, and BindingDB datasets, we set them to 500, 500, and 300, respectively, because the Davis and KIBA datasets tended to stabilize around 500 epochs, while the BindingDB dataset showed no significant improvement after 300 epochs. Similarly, different datasets also have different batch sizes. For batch size, we tried 32, 64, 128, 256. Finally, 256, 128, 128 were selected as the batch sizes for training and testing on the Davis, KIBA, and BindingDB datasets, respectively. We observed that a larger batch size improved training stability for the Davis dataset, while for KIBA and BindingDB, a batch size of 128 offered a better balance between memory efficiency and gradient update effectiveness.

### 3.3. Evaluation Metrics

The performance of MGF-DTA is evaluated on the DTA datasets using Mean Squared Error (MSE), Mean Absolute Error (MAE), Pearson Correlation Coefficient (Pearson), Spearman’s Rank Correlation Coefficient (Spearman), the Concordance Index (CI) [31], and the Modified Squared Correlation Coefficient ($r_{m}^{2}$).

MSE measures the average of the squared differences between predicted values and true values. Its formula is

(21) $$\mathrm{MSE}=\frac{1}{n}\sum_{i=1}^{n}{\left(y_{i}-{\hat{y}}_{i}\right)}^{2}$$

where n is the number of samples, $y_{i}$ is the true value, and ${\hat{y}}_{i}$ is the predicted value. MAE computes the average of the absolute differences between predicted values and true values. Its formula is

(22) $$\mathrm{MAE}=\frac{1}{n}\sum_{i=1}^{n}\left|y_{i}-{\hat{y}}_{i}\right|$$

The Pearson coefficient measures the degree of linear correlation between predicted values and true values, with a range of [−1,1]. Its formula is

(23) $$\mathrm{Pearson}=\frac{\varphi (p,y)}{\varphi (p)\varphi (y)}$$

where φ(p,y) is the covariance between the predicted value and the label, φ(p) is the standard deviation of the predicted value, and φ(y) is the standard deviation of true value. The Spearman coefficient assesses the monotonic correlation in ranking between predicted values and true values. Its formula is

(24) $$\mathrm{Spearman}=1-\frac{6\sum_{i=1}^{n}d_{i}^{2}}{n(n^{2}-1)}$$

where $d_{i}$ is the difference in ranks between the true value and the predicted value for the *i*-th sample. CI is used to evaluate the model’s ability to rank the binding affinities of drug–target pairs. Its formula is

(25) $$\mathrm{CI}=\frac{1}{Z}\sum_{\delta_{i}>\delta_{j}}h\left(y_{i}-y_{j}\right)$$

where $y_{i}$ is the predicted value of $\delta_{i}$, $y_{j}$ is the predicted value of $\delta_{j}$, Z is the normalization constant, and h(x) is the step function:

(26) $$h\left(x\right)=\left\{\begin{array}{cc} 0, & x<0\\ 0.5, & x=0\\ 1, & x>0 \end{array}\right.$$

The $r_{m}^{2}$ index assesses the external prediction performance of the model. An $r_{m}^{2}$ value close to 1 indicates good external prediction performance and high reliability, as defined below.

(27) $$r_{m}^{2}=r^{2}\left(1-\sqrt{r^{2}-r_{0}^{2}}\right)$$

where $r^{2}$ and $r_{0}^{2}$ are the squared correlation coefficients with and without intercept.

## 4. Conclusions

In this work, we introduce MGF-DTA, a multi-granularity fusion model for drug–target binding affinity prediction. The main contributions of this work include the integration of multi-source drug features via gated fusion, the enhancement of protein representations through residual fusion, and the design of a hierarchical attention mechanism to achieve multi-granularity interactive learning between drugs and targets. Furthermore, MGF-DTA demonstrates strong performance across various affinity datasets. Finally, case studies validate its good generalization capability, highlighting its potential as a valuable technique for drug reuse and screening.

In future work, building upon the existing multi-modal fusion framework, we will further enhance the modeling capability for protein and drug molecular representations, and explore more advanced strategies for feature interaction and fusion. Specifically, we will investigate how to integrate protein 3D structural information into the current sequence-based representation system, aiming to more accurately characterize the binding region. In addition, we will develop a lightweight cross-modal feature interaction module and explore knowledge distillation techniques to reduce model complexity and inference time while preserving predictive performance. Finally, while the k-mer+PCA approach with residual fusion has achieved performance improvement in drug–target affinity prediction, the majority of protein sequences in DTA datasets still fall below the maximum length limit of ESM-2. Further exploration is needed to compensate for the inherent limitations of ESM-2 and thereby enhance prediction performance.

## Figures and Tables

**Table 1:** Performance comparison of MGF-DTA with and without fingerprint information integration on the three benchmark datasets.

Dataset	Feature Composition	Fusion Method	MAE ↓	MSE ↓	Pearson ↑	Spearman ↑	CI ↑
Davis	ChemBERTa-2	-	0.214	0.192	0.878	0.699	0.904
	ChemBERTa-2 +Fingerprints	Gated fusion	0.211 *	0.186 *	0.883 *	0.706 *	0.909 *
KIBA	ChemBERTa-2	-	0.213	0.152	0.886	0.881	0.887
	ChemBERTa-2 +Fingerprints	Gated fusion	0.199 *	0.139 *	0.892 *	0.886 *	0.894 *
BindingDB	ChemBERTa-2	-	0.430	0.555	0.879	0.836	0.872
	ChemBERTa-2 +Fingerprints	Gated fusion	0.404 *	0.547 *	0.880	0.839 *	0.874 *

The down arrows ↓ denote that a lower value indicates better performance, while the up arrows ↑ denote that a higher value indicates better performance. The asterisk * indicates significance.

**Table 2:** Comparative analysis of drug feature fusion methods on the three benchmark datasets.

Dataset	Methods	MAE ↓	MSE ↓	Pearson ↑	Spearman ↑	CI ↑
Davis	Concat	0.250 ^a^	0.203 ^a^	0.870 ^a^	0.690 ^a^	0.897 ^a^
	Weighted Fusion	0.211 ^b^	0.187 ^b^	0.882 ^b^	0.696 ^b^	0.903 ^b^
	Cross-Attention	0.215 ^c^	0.188 ^b^	0.880 ^b^	0.693 ^c^	0.900 ^c^
	Gated Fusion (ours)	0.211 ^b^	0.186 ^b^	0.883 ^b^	0.706 ^d^	0.909 ^d^
KIBA	Concat	0.208 ^a^	0.143 ^a^	0.888 ^a^	0.883 ^a^	0.892 ^a^
	Weighted Fusion	0.206 ^b^	0.143 ^a^	0.889 ^a^	0.883 ^a^	0.891 ^a^
	Cross-Attention	0.226 ^c^	0.171 ^b^	0.866 ^b^	0.863 ^b^	0.879 ^b^
	Gated Fusion (ours)	0.199 ^d^	0.139 ^c^	0.892 ^c^	0.886 ^c^	0.894 ^c^
BindingDB	Concat	0.421 ^a^	0.552 ^a^	0.880 ^a^	0.836 ^a^	0.873 ^a^
	Weighted Fusion	0.413 ^b^	0.549 ^b^	0.880 ^a^	0.837 ^a^	0.873 ^a^
	Cross-Attention	0.415 ^b^	0.551 ^a^	0.878 ^b^	0.832 ^b^	0.870 ^b^
	Gated Fusion (ours)	0.404 ^c^	0.547 ^c^	0.881 ^a^	0.839 ^c^	0.874 ^a^

The down arrows ↓ denote that a lower value indicates better performance, while the up arrows ↑ denote that a higher value indicates better performance. Methods that share the same superscript letter within a column are not significantly different (*p* ≥ 0.05), while those with differing superscript letters exhibit statistically significant differences (*p* < 0.05).

**Table 3:** Performance comparison of MGF-DTA with and without k-mer information integration on the three benchmark datasets.

Dataset	Feature Composition	Fusion Method	MAE ↓	MSE ↓	Pearson ↑	Spearman ↑	CI ↑
Davis	ESM-2	-	0.211	0.186	0.883	0.706	0.909
	ESM-2+k-mer	Residual fusion	0.209 *	0.185	0.883	0.710 *	0.911 *
KIBA	ESM-2	-	0.199	0.139	0.892	0.886	0.894
	ESM-2+k-mer	Residual fusion	0.199	0.137 *	0.893	0.888 *	0.895
BindingDB	ESM-2	-	0.404	0.547	0.880	0.839	0.874
	ESM-2+k-mer	Residual fusion	0.403	0.542 *	0.883 *	0.840	0.875

The down arrows ↓ denote that a lower value indicates better performance, while the up arrows ↑ denote that a higher value indicates better performance. The asterisk * indicates significance.

**Table 4:** Comparative analysis of drug–target feature fusion methods on the three benchmark datasets.

Dataset	Methods	MAE ↓	MSE ↓	Pearson ↑	Spearman ↑	CI ↑
Davis	Concat	0.212 ^a^	0.184 ^a^	0.884 ^a^	0.705 ^a^	0.908 ^a^
	Bilinear Fusion	0.239 ^b^	0.199 ^b^	0.875 ^b^	0.702 ^b^	0.906 ^b^
	Weighted Fusion	0.213 ^a^	0.190 ^c^	0.880 ^c^	0.688 ^c^	0.897 ^c^
	Cross-Attention	0.212 ^a^	0.206 ^d^	0.868 ^d^	0.685 ^d^	0.895 ^d^
	Linear Attention	0.209 ^c^	0.185 ^a^	0.883 ^a^	0.710 ^e^	0.911 ^e^
	Hierarchical Attention (ours)	0.208 ^c^	0.183 ^a^	0.884 ^a^	0.714 ^f^	0.913 ^f^
KIBA	Concat	0.204 ^a^	0.138 ^a^	0.893 ^a^	0.884 ^a^	0.890 ^a^
	Bilinear Fusion	0.311 ^b^	0.251 ^b^	0.796 ^b^	0.783 ^b^	0.825 ^b^
	Weighted Fusion	0.211 ^c^	0.145 ^c^	0.888 ^c^	0.882 ^a^	0.889 ^a^
	Cross-Attention	0.259 ^d^	0.178 ^d^	0.862 ^d^	0.854 ^c^	0.869 ^c^
	Linear Attention	0.199 ^e^	0.137 ^a^	0.893 ^a^	0.888 ^d^	0.895 ^d^
	Hierarchical Attention (ours)	0.198 ^e^	0.132 ^e^	0.898 ^e^	0.891 ^e^	0.897 ^e^
BindingDB	Concat	0.451 ^a^	0.620 ^a^	0.867 ^a^	0.823 ^a^	0.864 ^a^
	Bilinear Fusion	0.494 ^b^	0.730 ^b^	0.837 ^b^	0.796 ^b^	0.848 ^b^
	Weighted Fusion	0.450 ^a^	0.626 ^c^	0.860 ^c^	0.811 ^c^	0.857 ^c^
	Cross-Attention	0.445 ^c^	0.610 ^d^	0.868 ^a^	0.829 ^d^	0.858 ^c^
	Linear Attention	0.403 ^d^	0.542 ^e^	0.883 ^d^	0.840 ^e^	0.874 ^d^
	Hierarchical Attention (ours)	0.400 ^e^	0.540 ^f^	0.883 ^d^	0.841 ^e^	0.876 ^e^

The down arrows ↓ denote that a lower value indicates better performance, while the up arrows ↑ denote that a higher value indicates better performance. Methods that share the same superscript letter within a column are not significantly different (*p* ≥ 0.05), while those with differing superscript letters exhibit statistically significant differences (*p* < 0.05).

**Table 5:** Performance of MGF-DTA and other mainstream methods on Davis and KIBA datasets.

Method	Davis			KIBA		
	MSE ↓	CI ↑	$R_{m}^{2}$↑	MSE ↓	CI ↑	$R_{m}^{2}$↑
DeepDTA	0.261	0.878	0.630	0.194	0.863	0.673
GraphDTA	0.241	0.869	0.632	0.177	0.868	0.733
MGraphDTA	0.217	0.879	0.673	0.148 ^a^	0.894 ^a^	0.775 ^a^
AttentionDTA	0.215	0.879	0.663	0.167	0.880	0.732
TF-DTA	0.231	0.886	0.670	0.177	0.877	0.734
LLMDTA	0.226	0.884	0.717	0.162	0.872	0.768
SMFF-DTA	0.206	0.897	0.733	0.151	0.894	0.780
PMMR	0.194 ^a^	0.910 ^a^	0.751 ^a^	0.163	0.880	0.764
MGF-DTA (Ours)	0.183 ^b^	0.913 ^b^	0.779 ^b^	0.132 ^b^	0.897 ^b^	0.784 ^b^

The down arrows ↓ denote that a lower value indicates better performance, while the up arrows ↑ denote that a higher value indicates better performance. Methods that share the same superscript letter within a column are not significantly different (*p* ≥ 0.05), while those with differing superscript letters exhibit statistically significant differences (*p* < 0.05).

**Table 6:** Performance of MGF-DTA and PMMR on BindingDB dataset.

Method	MSE ↓	CI ↑	$R_{m}^{2}$↑
PMMR	0.555	0.872	0.760
MGF-DTA (Ours)	0.540 *	0.876 *	0.765 *

The down arrows ↓ denote that a lower value indicates better performance, while the up arrows ↑ denote that a higher value indicates better performance. The asterisk * indicates significance.

**Table 7:** Performance of MGF-DTA and other mainstream methods on TDC-DG dataset.

Method	Pearson ↑
MGF-DTA (Ours)	0.591 ^a^
OTTER-KNOWLEDGE	0.588 ^b^
ProBertMorgan	0.538
MMD	0.433
CORAL	0.432
ERM	0.427
MTL	0.425
GroupDRO	0.384

The up arrows ↑ denote that a higher value indicates better performance. Methods that share the same superscript letter within a column are not significantly different (*p* ≥ 0.05), while those with differing superscript letters exhibit statistically significant differences (*p* < 0.05).

**Table 8:** The ablation experiment results obtained on the Davis dataset.

Model	A	B	C	MSE ↓	CI ↑	Spearman ↑
Model-1	–	–	–	0.192 ^a^	0.904 ^a^	0.699 ^a^
Model-2	√	–	–	0.186 ^b^	0.909 ^b^	0.706 ^b^
Model-3	√	√	–	0.185 ^b^	0.911 ^c^	0.710 ^c^
Model-4 (MGF-DTA)	√	√	√	0.183 ^c^	0.913 ^d^	0.714 ^d^

A: Gated fusion of drugs. B: Residual fusion of proteins. C: Hierarchical fusion mechanism. The down arrows ↓ denote that a lower value indicates better performance, while the up arrows ↑ denote that a higher value indicates better performance. Methods that share the same superscript letter within a column are not significantly different (*p* ≥ 0.05), while those with differing superscript letters exhibit statistically significant differences (*p* < 0.05).

**Table 9:** Case study on samples from the Metz dataset.

Metz ID	Metz Value	Predicted Value (KIBA Score)
PRKG1	8.1	14.28
PRKAA1	8.0	13.64
MINK	7.2	12.77
CDK2	7.0	12.57
DYRK4	6.6	12.10
PIM2	6.4	12.79
STK6	6.0	11.66
SRC	5.8	11.50
NTRK2	5.6	11.41
CAMK2D	5.3	11.28
SGK	5.1	11.19
KIAA1811	5.0	11.17
GSK3B	4.7	11.25
ACK1	4.6	10.53
ABL1	4.3	10.51

## Data Availability

The data presented in this study are openly available in [GitHub] at https://github.com/fdmsz/MGF-DTA, accessed on 21 December 2025.
